# Long-term fertilization alters chemically-separated soil organic carbon pools: Based on stable C isotope analyses

**DOI:** 10.1038/srep19061

**Published:** 2016-01-11

**Authors:** Xiaolin Dou, Ping He, Xiaoli Cheng, Wei Zhou

**Affiliations:** 1 Institute of Agricultural Resources and Regional Planning, Chinese Academy of Agricultural Sciences (CAAS), Beijing 100081, P.R. China; 2 Wuhan Botanical Garden, Chinese Academy of Sciences (CAS), Wuhan 430074, P.R. China

## Abstract

Quantification of dynamics of soil organic carbon (SOC) pools under the influence of long-term fertilization is essential for predicting carbon (C) sequestration. We combined soil chemical fractionation with stable C isotope analyses to investigate the C dynamics of the various SOC pools after 25 years of fertilization. Five types of soil samples (0–20, 20–40 cm) including the initial level (CK) and four fertilization treatments (inorganic nitrogen fertilizer, IN; balanced inorganic fertilizer, NPK; inorganic fertilizer plus farmyard manure, MNPK; inorganic fertilizer plus corn straw residue, SNPK) were separated into recalcitrant and labile fractions, and the fractions were analysed for C content, C:N ratios, δ^13^C values, soil C and N recalcitrance indexes (RIC and RIN). Chemical fractionation showed long-term MNPK fertilization strongly increased the SOC storage in both soil layers (0–20 cm = 1492.4 gC m^2^ and 20–40 cm = 1770.6 gC m^2^) because of enhanced recalcitrant C (RC) and labile C (LC). The 25 years of inorganic fertilizer treatment did not increase the SOC storage mainly because of the offsetting effects of enhanced RC and decreased LC, whereas no clear SOC increases under the SNPK fertilization resulted from the fast decay rates of soil C.

Soil organic carbon (SOC) plays a positive role in soil fertility, soil sustainability and crop yield in agricultural ecosystems^1^,^2^. Even a small change in SOC storage can greatly affect atmospheric carbon dioxide (CO_2_) concentrations^3^. Fertilizer application has been widely used as a common agricultural management strategy to promote soil carbon (C) sequestration^4^,^5^, which could directly or indirectly increase the SOC inputs and thereby influence nutrient availability and soil turnover^6^. For example, inorganic nitrogen (N) fertilizer may indirectly enhance the SOC storage by increased crop residue input to soils^1^,^7^, whereas organic manure application can influence soil organic matter (SOM) through the direct inputs of processed organic materials to soils^8^,^9^.

To date, positive^7^,^10^,^11^, negative^12^, and no clear effects^9^,^13^ have been reported of fertilization on soil C sequestration in agroecosystems. These inconsistencies may be explained by the fact that increased SOM input from fertilizers may be offset by the soil C loss from various soil fractions, resulting in zero accumulation of SOC, or even a negative deficit^8^,^14^. For example, previous studies have indicated that inputs of N into soil can enhance the decomposition of labile/light soil fractions, but inhibit the decomposition of recalcitrant/heavy soil fractions, which may lead to no net changes in total SOC pool^15^,^16^. Therefore, insight is urgently needed into soil C dynamics under long-term fertilization.

Detecting the soil C dynamics in ecosystems is generally difficult, because SOM is a complex compounds that consists of labile and recalcitrant pools^17^,^18^. Turnover times of the two fractions can differ considerably because of the differences in their chemical and physical stability^3^,^4^,^19^. For instance, the labile C (LC) is readily decomposable and sensitive and responds quickly to changes in management practices, while recalcitrant C (RC) is a major C store with a more stable fraction^5^,^19^. Originally, C_4_ (δ^13^C *ca.* −12‰) and C_3_ (δ^13^C *ca*. −28‰) plants may produce detritus with different ^13^C/^12^C ratios because of their differences in using C isotopes^20^. The relative contribution of new SOC vs. old SOC can be estimated based on the mass balance of C isotope contents, and thus it becomes possible to estimate SOM turnover rate *in situ*^20^,^21^. In fact, this technology has increasingly been applied to important issues such as land-use change and reforestation with no shift in photosynthetic strategy in the ecosystems^3^,^19^,^20^. These studies have successfully revealed the dynamics of SOC pools/fractions under global environmental change. In the present study, soil C turnover was quantified using δ^13^C abundance based on the changes in decomposition level following changes in external conditions such as 25 years of fertilization^3^,^4^. In short, the use of naturally abundant stable C isotopes combined with SOM chemical fractionation techniques are an approach that can better quantify SOM dynamics under long-term fertilization in agro-ecosystems^3^,^19^.

Black soils, typical Mollisols with a rich organic matter content, are the most fertile and productive soils in China and are mainly distributed in northeast regions^22^. In recent decades, the productivity of the black soils has been in decline as a result of non-sustainable agricultural practices^23^. In the agricultural tillage system common to China, aboveground crop residue is usually removed for energy use or as livestock feed, which could result in a decline of SOM, a depletion of C stocks, deterioration of soil structure, and serious soil erosion. Therefore, an abundance of inorganic and organic fertilizers (e.g., N fertilizer and manure) are applied in cropland to improve the SOM quality and quantity and to help increase the crop yield^22^. A long-term field experiment has been conducted with a continuous corn cropping system since 1989 in Gongzhuling, Jilin Province of China, enabling the dynamics of SOC pools to be explored under long-term fertilization. In this study, we hypothesise that 25 years of fertilization would significantly change SOC storage and C turnover rate in a black soil of northeast China. To test this hypothesis, we measured the δ^13^C, C and N content in the soil organic pools (labile and recalcitrant pools) and plant samples from the top 40 cm of different fertilizer-treated soils.

## Results

### The soil physicochemical properties, plant biological traits and soil δ^13^C

Table 1 shows the soil total C and N content, soil bulk density and pH under different types of fertilizer application. Both total C and N contents were greater in MNPK- and SNPK-treated soils and lower in IN- and NPK-treated soils compared with CK (Table 1). The soil bulk density was significantly higher in IN- and NPK-treated soils than in MNPK- and SNPK-treated soils and CK within the top layer (0–20 cm), whereas no significant difference in soil bulk density of the deep layer (20–40 cm) was observed among fertilization treatments (Table 1). The lowest pH values (pH = 6.3 and 6.4) occurred in IN- and NPK-treated soils (Table 1). The δ^13^C values of the leaf and roots varied from −13.36 ‰ to −16.23‰ and from −12.67 ‰ to −14.35‰, respectively, in the corn-planted field, which were typical of C_4_ plants (Table 2). The C:N ratios in the leaf and roots of the corn decreased in the following order: SNPK > MNPK > NPK > IN-treated soils (Table 2).

Long-term fertilization strongly altered the δ^13^C values of the soil organic pool and RC pool with, as expected, less negative δ^13^C values occurring in fertilized soils compared with the initial soils due to the C_4_ residue inputs at both 0–20 and 20–40 cm depth (Table 3). The least negative δ^13^C values in the SOC pool occurred in SNPK-treated soils, whereas the least negative δ^13^C values in the RC pool occurred in MNPK- and SNPK-treated soils (Table 3). Overall, the δ^13^C values of the RC pool decreased in the following order: SNPK/MNPK > NPK > IN > CK-treated soils in both soil layers (Table 3).

### C content and storage of total soil organic pools, soil LC and RC pools

**Figure 1:**
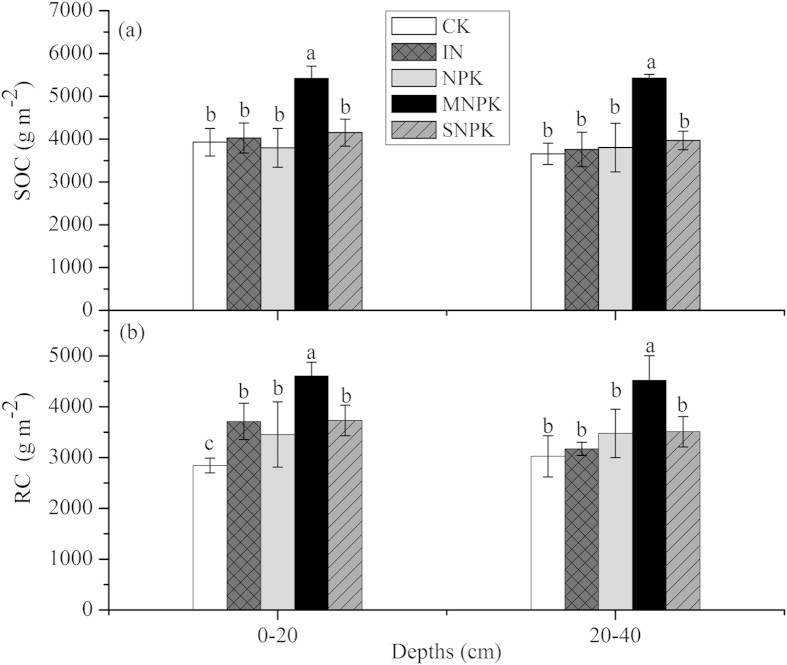
Changes in SOC (**a**) and RC (**b**) storage (mean ± SE, n = 3) under different long-term fertilization treatments at two soil depths. Values followed by a different lowercase letter over the bars of root indicate statistically significant differences at *P* < 0.05 among fertilization treatments.

Long-term fertilization significantly affected the soil C content and C:N ratios in the total organic C pool, RC pool and LC pool (*P* < 0.001), except for C:N ratios in the total organic C pool. In contrast, soil depths altered the C:N ratios of the soil organic pool and C content of the LC pool (*P* < 0.05; Table 4). Overall, the greatest SOC content of the total organic C pool and RC and LC pools was found in MNPK-treated soils, followed by SNPK and then by inorganic fertilizers (Table 4). The SOC content of the total organic C pool and LC pool was greater in MNPK- and SNPK-treated soils and lower in IN- and NPK-treated soils than CK in the top layer. However, SOC storage of the total organic C pool was greatest in MNPK-treated soils (5423.3 gC m^2^, on average) compared with other treatments in both soil layers (Table 4; Fig. 1). The SOC content and storage of the RC pool was greater in all fertilized soils than in CK with the decreasing order as follows: MNPK > SNPK > NPK/IN > CK-treated soils (Table 4; Fig. 1). Moreover, SOC content declined from the top soil (0–20 cm) to the deep layers (20–40 cm) under organic fertilizer treatments (MNPK and SNPK). The C:N ratios of the RC pool decreased in the following order: NPK > IN > SNPK > CK > MNPK-treated soils, whereas the C:N ratios of the LC pool decreased in the following order: SNPK > MNPK > NPK > IN > CK-treated soils in both soil layers (Table 4).

### Soil organic C turnover

By applying the mass balance of a stable isotope, soil organic C was partitioned into new and old C (i.e., older than 25 years). Application of long-term fertilization stimulated both new C input and the decay rate of the old C, relative to CK (*P* < 0.01; Table 5). The new C inputs into SOC pools were greatest in SNPK-treated soils with a new C proportion of 22.51% for the top soil and 28.35% for the deep soil layers, followed by MNPK-treated soils with a new C proportion of 16.21% for the top soil and 20.00% for the deep soil layers (Table 5). In contrast, the proportion of new C in the SOC was 8.20–10.30% for the top soil and 14.74–18.77% for the deep soil in inorganic fertilizer-treated soils (IN and NPK). Accordingly, the fastest decay rates of the old C were found in SNPK-treated soils and the lowest in IN- and NPK-treated soils. In general, decay rates for the old C in the deep layer were faster than in the top layer in the fertilized soils (Table 5).

### Recalcitrance index for C and N

**Figure 2:**
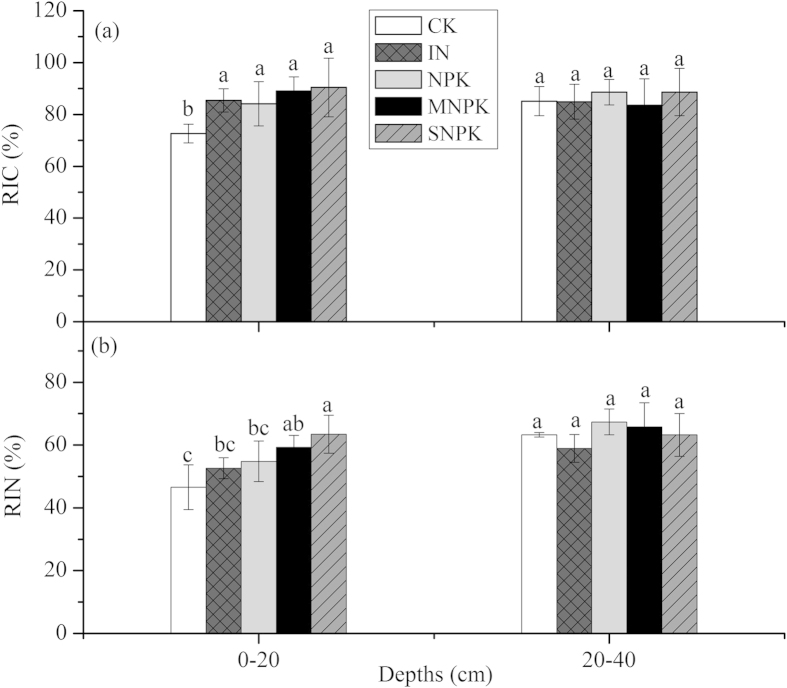
Variations in recalcitrance indices with depth for (**a**) carbon (RIC) and (**b**) nitrogen (RIN) under different long-term fertilization treatments at two soil depths (mean ± SE, n = 3). Values followed by a different lowercase letter over the bars of root indicate statistically significant differences at *P* < 0.05 among fertilization treatments.

Long-term fertilization significantly increased the RIC and RIN ratios in the fertilized soils compared with CK in the top soil layer (Fig. 2). The long-term SNPK fertilizer treatment resulted in the highest RIC (90.44%) and RIN (63.45%) ratios of the all treatments (Fig. 2). In contrast, the lowest RIC (84.16%) and RIN (52.59%) ratios appeared in the IN- and NPK-treated soils. No significant differences were found in RIC and RIN ratios among various fertilization treatments in the deep soil layers (Fig. 2).

## Discussion

The results of this study showed that 25 years of fertilization significantly altered soil C dynamics, which is consistent with our hypothesis. The greatest SOC content and storage was found in MNPK-treated soils, followed by SNPK and then by inorganic fertilizers in the organic C pool (Table 4), which is consistent with the previous study that SOC was the highest in NPK fertilizers combined with organic matter, followed by NPK on corn land^9^. Jiang G *et al.* (2014) drew the conclusion that SOC sequestration potential would mostly be a net source of CO_2_ under the condition of no future fertilizer input in northern China^24^. However, application of manure or straw to soils was able to improve the C sequestration potential, but inorganic fertilizers were not^24^. This supports our similar findings. Generally, RC has been shown to be a major C store^3^ and our results also showed that RC content and storage were greater in all fertilized soils than in the initial soils (Table 4; Fig. 1). This suggests that long-term fertilization had a positive overall impact on soil RC accumulation, regardless of the application of inorganic or organic fertilizers. Overall, SOC content declined from the top soil to deep layers in the soil organic pools and its chemical fractions across fertilization treatments (Table 4). This was because the decay rates of soil C in the deep soil were faster than those in the top layer (Table 5).

Previous studies have shown that the amount of labile C is proportional to the SOM input to the soil, thus LC is more affected than RC by land management practices, and thereby LC responds quickly and sensitively to the changes in SOM pools^17^. Our results confirm this observation, especially in N- and NPK-treated soils, in which the SOC content of the LC pool maintained the same trend as that in the total organic pool (Table 4). We found that there were no net changes in SOC storage relative to CK after application of long-term N and NPK fertilizers (Fig. 1), which indicated that long-term IN and NPK fertilizers decreased the SOC content of organic pools (Table 4), but significantly increased soil density in the 0–20 cm layer^25^ (Table 1). Our results confirmed previous findings that 25 years of continuous application of inorganic fertilizer was not capable of increasing the total SOC compared with the control^8^,^23^. One possible explanation is that inorganic fertilizers were insufficient for preserving SOC levels under conventional tillage management because of no above-ground crop residues returning to the soil^24^, although inorganic fertilizers may indirectly enhance SOM by increasing plant biomass and C return to soils^23^. Furthermore, the simple addition of inorganic IN and NPK fertilizers led to soil acidification (Table 1), which correspondingly affected soil microbial activity and labile C such as microbial biomass C^26^. Moreover, a positive correlation was found between soil pH and microbial biomass C^26^. Therefore, the other possible explanation for the finding of no clear increases of SOC storage is that soil acidification resulting from inorganic fertilizers affected the SOC pool, such as soil labile C pool^13^,^26^. We also concluded that no apparent changes in SOC storage of total organic pools occurred in IN- and NPK-treated soils, mainly owing to the offsetting effects between enhanced SOC in the recalcitrant pool and decreased SOC in the labile pool (Table 4; Fig. 1).

In contrast, application of a long-term MNPK fertilizer strongly increased the SOC storage by 1492.4 gC m^2^ in the top layer and 1770.6 gC m^2^ in the deep layer on average, whereas it increased the RC storage by 1759.85 gC m^2^ in the top layer and 1499.67 gC m^2^ on average in the deep layer (Fig. 1). This supports the fact that long-term addition of manure combined with inorganic fertilizers significantly increased SOC content^8^. The δ^13^C of SOC seemed to be more enriched than that of CK at both sampled depths in MNPK-treated soils due to a higher contribution of C_4_ residues (Table 3), which provided evidence that the SOC storage substantially increased not only in the top layer but also in the deep layer in the corn-planted field (Fig. 1). This was because roots dominated the inputs of SOC, such as root biomass and exudates^27^, and the larger corn roots were distributed mainly in the 20–30 cm soil layer at the experimental site^28^. In contrast, SOC in the top layer (0–10 cm) was usually rapidly lost, possibly through soil respiration and incomplete decomposition of SOM in residues^27^. In the present study, the increased SOC storage in MNPK-treated soils was mainly caused by C accumulation in soils via manure inputs, given the high SOC content of about 112 g kg^−1^ at the experimental site^23^. Additionally, the addition of farmyard manure as a high-quality organic resource with low C:N ratios of 26:1^25^ is likely to result in lower C:N ratios and a rapid loss of C during decomposition in the MNPK-treated soils^29^,^30^. Indeed, relatively fast decay rates of old C occurred in the MNPK-treated soils in our study (Table 5). As we know, SOC storage represents the net change of organic matter inputs to soil and losses through SOM decomposition^1^,^31^. Therefore, our results indicate that the positive effect of manure addition through the return of belowground biomass or the direct amendments of organic manure with a high SOC content was not offset by the soil C decomposition in MNPK-treated soils^12^,^30^. Additionally, we conclude that application of long-term MNPK fertilization significantly enhanced the organic C pool largely because of the increased SOC in both recalcitrant and labile pools (Table 4; Fig. 1). Thus, MNPK fertilizer was shown to be the most effective measure for soil C sequestration in the longer term.

Crop straw return is recommended as an important management practice in the agricultural sector^32^,^33^. Many studies have reported that the addition of crop straw to soils could help improve the soil C sequestration via its favorable effects on soil physicochemical properties in both the long and short term^33^,^34^. In our study, long-term SNPK fertilization caused no significant increases in SOC content and storage of the total organic C pool and LC pool, but increased RC storage on average by 886.13 gC m^2^ in the top layer and 484.35 gC m^2^ in the deep layer (Fig. 1). In contrast, Zhu *et al.* (2015) showed that SOC and labile organic C content were higher under the straw return treatments and inorganic fertilizer addition compared with the no straw addition treatment at 0–21 cm soil depth after a 2-year field experiment^33^. Zhang *et al.* (2014) found that 4 years of straw addition to soils was beneficial for the accumulation of SOC, and decreased the SOC losses from conventional tillage^32^. The differences between our results and these studies may be caused by the differences in experimental time. The soil LC fraction is dominated by newly incorporated plant-derived materials and shows a rapid response to straw addition in the early stage, and thus shows an initial increasing trend of SOC^33^. However, our results further indicated that application of SNPK fertilizer would eventually result in no significant SOC increases but enhance soil C storage in the RC pool over longer experimental time periods. Generally, recalcitrant C was resistant to decomposition and had longer turnover time compared with labile C^17^. Therefore, the results indicated that no significant increases in SOC of the total organic pool may be attributed to the rapid soil C turnover of labile pools. Indeed, the fastest decay rates of old C of SOC pool were found in SNPK-treated soils (Table 5). However, straw is considered a low-quality organic resource with a high C:N ratio of 66:1^25^,^29^, and thus has a slow decomposition rate^35^. Interestingly, Chivenge *et al.* (2011)^29^ showed that straw decomposed slowly, but the addition of N fertilizers could negate some effects of this type of low-quality organic resource. Our results further proved that corn straw combined with inorganic fertilizers could accelerate the soil C turnover when compared with the simple addition of inorganic fertilizers or straw alone.

Unhydrolyzable organic matter is perceived as the recalcitrant fraction, and amount of unhydrolyzable organic matter has been used to measure the inactive organic pool^3^,^18^. The results obtained in the recalcitrance analysis suggest that long-term fertilization leads to major changes in the biochemical quality of SOM. We found that the RIC values were higher in the fertilized soils than in CK in the top layer (Fig. 2). This result was expected because application of inorganic and organic fertilizers should result in an increase in the recalcitrant C inputs produced by crop residue input to soils or by the direct inputs of organic materials into the soil^1^,^36^. Our stable isotopic analysis further confirmed that the δ^13^C abundance in the organic pool of the fertilized soils was enriched relative to CK soils (Table 3), owing to the higher contribution of C_4_ residues. Additionally, the RIN values were higher in MNPK- and SNPK-treated soils than in IN- and NPK-treated soils and CK in the top layer (Fig. 2), possibly because of the higher level of soil C and N mineralisation of labile pools in organic fertilizer-treated soils^9^,^23^. Moreover, more N might be retained because of the higher C supply (higher RIC) to the MNPK- and SNPK-treated soils and thus enhances the recalcitrant N proportions^37^.

## Materials and Methods

### Site descriptions and experimental design

A long-term fertilization experiment presented for monitoring black soil fertility and fertilizer efficiency with monoculture maize (*Zea mays L.*) has been conducted since 1989 at Gongzhuling, Jilin Province, China (124°48′33″E, 43°30′23″N)^23^,^25^. This region has a north temperate and semi-humid climate with an annual average temperature of 5.6 °C. The annual precipitation is approximately 562 mm, 80% of which falls between June and September^25^. The soil is a clay loam [Typic Hapludoll (Mollisol) in USDA Soil Taxonomy] developed from Quaternary loess-like sediments with 39% sand, 30% silt and 31% clay at the beginning of the experiment^23^. A randomized complete block design was used with three replicates in this long-term experiment with each replicate plot covered 130 m^2^. The experiment included five treatments: (1) Initial soils (CK); (2) inorganic nitrogen fertilizer at the rate of 165 kg N ha^−1^ (IN); (3) balanced inorganic fertilizers at 165 kg N ha^−1^, 82.5 kg P_2_O_5_ ha^−1^, and 82.5 kg K_2_O ha^−1^ (NPK); (4) balanced inorganic fertilizers at 50 kg N ha^−1^, 82.5 kg P_2_O_5_ ha^−1^, and 82.5 kg K_2_O ha^−1^ plus farmyard manure at the rate of 2.3 × 10^4^ kg ha^−1^ (MNPK), and (5) balanced inorganic fertilizers at 112 kg N ha^−1^, 82.5 kg P_2_O_5_ ha^−1^, and 82.5 K_2_O kg ha^−1^ plus corn straw residue at the rate of 7.5 × 10^3^ kg ha^−1^ (SNPK)^25^. The N contents in corn straw and farmyard manure were 7.0 and 5.0 g kg^−1^, respectively, and thus the total N application rates for IN, NPK, SNPK, and MNPK treatments were kept at 165 kg ha^−1^ 25. The organic C content of farmyard manure (mostly, pig manure) was about 112 g kg^−1^ 23; the δ^13^C of farmyard manure was measured with a value of −21.59‰. The sources of inorganic N, P, and K fertilizers were urea, triple superphosphate (TSP) and muriate of potash (MoP)^25^. The application of fertilizers was approximately 10 cm of soil depth. One third of the urea and total amounts of TSP and MoP were applied as a basal dose. The remaining two thirds of the urea was used for side dressing at the corn jointing stage, whereas the chopped corn straw was also applied in the SNPK plots with the top 25 cm of soil at that time every year^25^. The farmyard manure was applied in the MNPK plots after corn harvesting in autumn each year^25^. Corn was sown in late April and harvested in late September. Aboveground plant residues were removed at harvest. Prior to the long-term experiment, the field had been continuously cultivated corn for some years, and then was homogenized by growing corn for 3 years without fertilizer application^23^. The soil physiochemical properties (pH, bulk density, C and N content) were shown in Table 1. The pH and bulk density of soil were measured as previously described by Song *et al.* (2015)^25^.

### Field sample collection and soil fractionations

In August 2014, we randomly placed three sub-plots (2 m × 2 m) around the corn rhizosphere within each treatment plot; the distances between the sub-plots were approximately 5 m. Soil samples from each treatment plot were collected at 0–20 cm and 20–40 cm soil depths using a 5-cm diameter stainless steel soil corer. Newly produced corn leaves were collected in each treatment plot. Root sampling blocks were excavated within a 30 × 30 cm quadrant at a soil depth of 0–20 cm and then were washed clean carefully; leaves and roots were oven dried to a constant weight at 65 °C in the laboratory to prepare for determination. The soil samples were air-dried, after which the large roots and stones were removed by hand.

Soil labile and recalcitrant C were determined following the acid hydrolysis procedure described by Cheng *et al.* (2008)^17^ and Rovira and Vallejo (2002)^18^. Subsamples of soil were treated with 1 N HCl for 24 h at room temperature to remove any carbonate. This fraction was interpreted to be soil organic matter (SOM) pool. Approximately 500 mg of the SOM sample was hydrolyzed with 20 ml of 5 N H_2_SO_4_ for 30 min at 105 °C in sealed Pyrex tubes. The hydrolysate was recovered by centrifugation and decantation. The residue was washed with 20 ml of water, and the washing was added to the hydrolysate. This hydrolysate was interpreted to be Labile Pool (I). The residue was dried at 60 °C. The remaining residue was hydrolyzed with 2 ml of 26 N H_2_SO_4_ overnight at room temperature while being continuously shaken. Then, water was added to dilute the acid to 2 N and the sample was hydrolyzed for 3 h at 105 °C with occasional shaking. The hydrolysate was recovered by centrifugation and decantation. The residue was washed with 20 ml of water, and the washing was added to the hydrolysate. This hydrolysate was understood to be Labile Pool (II). Labile Pool (I) was added to Labile Pool (II) to obtain the total labile pool. The remaining residue was rinsed twice with water, transferred to a pre-weighed crucible, and dried at 60 °C. This fraction was interpreted to be the Recalcitrant Pool.

Similar to Rovira and Vallejo (2002)^18^, the recalcitrance indexes for C and N (RIC and RIN, respectively) were calculated by the following equations:


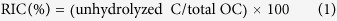



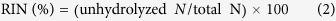


### C content and C isotope analyses

The above oven-dried plant materials and collected soil samples were ground to pass through 20-mesh (0.84 mm) sieves. The C and N content of the whole soil, plant materials (leaves and roots), soil organic pool, recalcitrant and labile pools were measured. The δ^13^C values were measured for soil plant materials, farmyard manure, soil organic pool, recalcitrant pool. Subsamples from all fractions were treated with 1 N HCl for 24 h at room temperature to remove any soil carbonates^3^, and weighed and analysed on an isotope ratio mass spectrometer (Thermo Finnigen, Delta-Plus, Flash, EA, 1112 Series, USA). The carbon isotope ratio of the soil fractions and plant materials was expressed as follows:


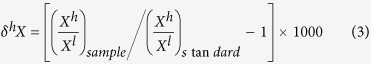


where *X* is carbon, *h* is the heavier C isotope, and *l* is the lighter C isotope. The CO_2_ samples were analysed relative to the internal working gas standards. The C isotope ratios (^13^C) are expressed as relative values to the Pee Dee Belemnite (δ^13^C = 0.0112372‰). The standards (acetanilide and spinach) were analysed after every ten samples; the analytical precision of the instrument was ± 0.13‰ for δ^13^C.

With respect to the plots of different fertilization treatments, the δ^13^C values of the SOM were used to calculate the proportion of new C (*f*_*new*_, i.e. the C derived from current corn residuals or fertilizers) and of old C (*f*_*old*_ = 1 − *f*_*new*_, soil C previous to fertilization, i.e., C in the initial soil) with a mass balance equation^38^:


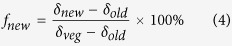


where *δ*_*new*_ is the δ^13^C values of SOC of the plant root-spheres soil under fertilization, *δ*_*old*_ is the δ^13^C values of organic C from initial soils, i.e. the soil samples previous to fertilization, and *δ*_*veg*_ is the δ^13^C values of the mixed plant materials of corn; Specially, *δ*_*veg*_ is the δ^13^C values of the mixed materials including plant and manure in MNPK treatment^3^,^20^.

Because the *δ*_*veg*_, *δ*_*new*_ and *δ*_*old*_ are independently measured, the standard errors (SE) of *f* associated with the use of the mass-balance approach can be calculated using partial derivatives^39^ as follows:


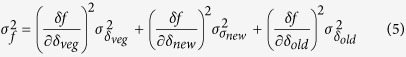


This equation can be reduced to:


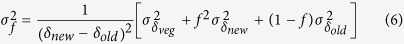


where 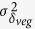, 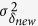 and 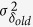 represent the variances of the mean *δ*_*veg*_, *δ*_*new*_ and *δ*_*old*_, respectively. The 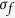 is the SE of the proportion (*f*) estimate^39^.

The decay rate constant (*k*) for the old C (i.e. the C of the organic matter before fertilization) of the soil organic pools was calculated based on Cheng *et al*. (2013)^3^:


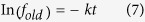


where *f*_old_=(1−*f*_new_) is the proportion of old C, *k* is the net relative decay rate constant for old C, and *t* is the age of fertilization (i.e. for 25 years).

### Statistics

The SOC content, C:N ratios, δ^13^C values, the new C input (*f*_new_), and the decay rate (*k*) of the old C of the soil organic pool for each treatment were calculated by averaging the three replicates for each sample plot. Before analysis, all variables were checked for a normal distribution and homogeneity of variance. Analysis of variance (ANOVAs) were performed to examine the differences in SOC level, the δ^13^C value, the C:N ratio of the organic soil, and the decay rate of the old C among fertilization treatments in relation to the soil depth (LSD; *P* = 0.05). An ANOVA of multiple comparisons was conducted to examine the effects of various fertilization treatments on total C and N, bulk density and pH of the whole soil, and the SOC level, the δ^13^C values, the C:N ratios of the soil organic pools (LSD; *P* = 0.05). All of the statistical analyses were performed using SPSS (version 16.0) and OriginPro (version 8.0) for Windows.

## Additional Information

**How to cite this article**: Dou, X. *et al.* Long-term fertilization alters chemically-separated soil organic carbon pools: Based on stable C isotope analyses. *Sci. Rep.* **6**, 19061; doi: 10.1038/srep19061 (2016).

## Figures and Tables

**Table 1:** Soil physiochemical properties under long-term fertilization in Gongzhuling, Jilin Province, China.

Treatments	TC (g kg^−1^)	TN (g kg^−1^)	BD (g cm^−3^)		pH
	0–20 cm	0–20 cm	0–20 cm	20–40 cm	0–20 cm
CK	16.50 ± 1.23^b^	2.11 ± 0.27^b^	1.17^b^	1.27^a^	7.6^a^
IN	15.87 ± 0.72^bc^	1.85 ± 0.06^b^	1.39^a^	1.24^a^	6.3^b^
NPK	14.59 ± 1.53^c^	1.84 ± 0.04^b^	1.34^a^	1.30^a^	6.4^b^
MNPK	23.47 ± 2.64^a^	2.87 ± 0.34^a^	1.21^b^	1.28^a^	7.4^a^
SNPK	17.04 ± 0.28^b^	2.15 ± 0.09^b^	1.20^b^	1.22^a^	7.7^a^

Data are expressed as mean ± SE, n = 3. Different letters indicate statistical significance at *P* < 0.05 among fertilization treatments. *Abbreviations*: TC, total carbon; TN, total nitrogen; BD, bulk density; CK, initial soil; IN, inorganic N fertilizer; NPK, balanced inorganic fertilizers of N, P and K; MNPK, balanced inorganic fertilizers plus farmyard manure; SNPK, balanced inorganic fertilizers plus corn straw residue.

**Table 2:** Stable carbon isotopic composition (δ^13^C) and C:N ratios of maize under long-term fertilization in Gongzhuling, Jilin Province, China.

Treatments	δ^13^C (‰)		C:N ratio	
	Leaf	Roots	Leaf	Roots
IN	−14.84 ± 0.23^a^	−13.35 ± 0.17^ab^	15.39 ± 2.13^c^	24.44 ± 3.67^b^
NPK	−13.36 ± 0.25^a^	−14.21 ± 0.21^b^	16.17 ± 0.86^bc^	28.33 ± 2.19^ab^
MNPK	−16.23 ± 0.34^b^	−14.35 ± 1.16^b^	16.95 ± 1.43^b^	28.45 ± 4.09^a^
SNPK	−14.76 ± 0.56^a^	−12.67 ± 0.33^a^	19.09 ± 3.08^a^	29.56 ± 2.01^a^

The abbreviations for fertilization treatments are the same as presented in Table 1. Data are expressed as mean ± SE, n = 3. Different letters indicate statistical significance at *P* < 0.05 among fertilization treatments.

**Table 3:** The δ^13^C values of soil (0–40 cm) organic pools under long-term fertilization.

	Depths (cm)	δ^13^C (‰)	
		Soil organic pool	Recalcitrant pool
Initial soils (CK)	0–20	−21.39 ± 0.88^c^	−21.15 ± 0.13^d^
	20–40	−22.18 ± 0.57^c^	−22.46 ± 0.44^c^
Treatments			
IN	0–20	−20.64 ± 0.22^b^	−19.38 ± 0.36^c^
	20–40	−20.66 ± 0.17^b^	−18.87 ± 0.61^b^
NPK	0–20	−20.76 ± 0.30^b^	−17.80 ± 1.51^b^
	20–40	−20.94 ± 0.60^b^	−17.28 ± 1.85^ab^
MNPK	0–20	−20.40 ± 0.03^b^	−15.58 ± 1.07^a^
	20–40	−20.80 ± 0.20^b^	−16.26 ± 1.44^a^
SNPK	0–20	−19.66 ± 0.42^a^	−16.65 ± 1.39^ab^
	20–40	−19.78 ± 0.24^a^	−16.35 ± 1.50^a^
Source of variation			
Fertilization		***	***
Depth		*	n.s.
Fertilization × Depth		n.s.	n.s.

The abbreviations for fertilization treatments are the same as presented in Table 1. Data are expressed as mean ± SE, n = 3. Different letters indicate statistical significance at *P* < 0.05 among fertilization treatments. Statistically significant differences are given after factorial ANOVA (n.s. not significant; *P < 0.05; **P < 0.01; ***P < 0.001).

**Table 4:** Soil C content and C:N ratios of soil organic pools (0–40 cm) under long-term fertilization.

	Depths (cm)	Soil organic pool		Recalcitrant pool		Labile pool	
		C (g kg^−1^)	C:N ratio	C (g kg^−1^)	C:N ratio	C (mg L^−1^)	C:N ratio
Initial soils (CK)	0–20	16.79 ± 1.38^b^	8.07 ± 0.71^ab^	12.17 ± 0.62^c^	12.69 ± 1.12^ab^	38.69 ± 3.31^b^	1.47 ± 0.02^c^
	20–40	14.39 ± 0.98^b^	9.22 ± 0.30^a^	11.91 ± 1.59^c^	12.86 ± 1.16^ab^	37.74 ± 4.24^b^	1.34 ± 0.02^c^
Treatments							
IN	0–20	14.49 ± 1.25^c^	7.71 ± 0.70^b^	13.36 ± 1.28^c^	13.48 ± 0.35^ab^	37.15 ± 1.66^bc^	1.68 ± 0.13^c^
	20–40	15.15 ± 1.63^b^	9.39 ± 0.78^a^	12.79 ± 0.52^bc^	13.51 ± 0.49^a^	35.06 ± 0.73^bc^	1.56 ± 0.21^bc^
NPK	0–20	14.16 ± 1.69^c^	8.25 ± 1.34^ab^	12.89 ± 2.40^c^	13.63 ± 1.98^a^	33.15 ± 1.12^c^	1.79 ± 0.24^c^
	20–40	14.63 ± 2.18^b^	10.15 ± 1.03^a^	13.38 ± 1.83^bc^	13.78 ± 0.67^a^	32.32 ± 0.96^c^	1.92 ± 0.21^b^
MNPK	0–20	22.40 ± 1.17^a^	8.34 ± 1.00^ab^	19.04 ± 1.12^a^	11.93 ± 0.55^b^	46.18 ± 3.46^a^	2.63 ± 0.41^b^
	20–40	21.20 ± 0.34^a^	9.61 ± 0.70^a^	17.68 ± 1.87^a^	12.18 ± 0.41^b^	43.91 ± 4.57^a^	2.72 ± 0.40^a^
SNPK	0–20	17.30 ± 1.33^b^	9.22 ± 0.11^a^	15.55 ± 1.25^b^	13.12 ± 0.75^ab^	40.34 ± 3.71^b^	3.10 ± 0.41^a^
	20–40	16.27 ± 0.88^b^	9.58 ± 0.69^a^	14.39 ± 1.23^b^	13.41 ± 0.34^a^	35.83 ± 0.92^bc^	3.07 ± 0.31^a^
Source of variation							
Fertilization		***	n.s.	***	***	***	***
Depth		n.s.	*	n.s.	n.s.	*	n.s.
Fertilization × Depth		n.s.	n.s.	n.s.	n.s.	n.s.	n.s.

The abbreviations for fertilization treatments are the same as presented in Table 1. Data are expressed as mean ± SE, n = 3. Different letters indicate statistical significance at *P* < 0.05 among fertilization treatments. Statistically significant differences are given after factorial ANOVA (n.s. not significant; *P < 0.05; **P < 0.01; ***P < 0.001).

**Table 5:** New C input (*f*_*new*_) and decay rate (*k*, yr^−1^) of old C of soil organic pools (0–40 cm) under long-term fertilization.

Treatments	Depths (cm)	*f*_*new*_ (%)	Decay rate (*k*) of old C
IN	0–20	10.30 ± 1.56^c^	0.004 ± 0.000^c^
	20–40	18.77 ± 2.21^b^	0.008 ± 0.001^b^
NPK	0–20	8.20 ± 1.08^d^	0.003 ± 0.000^c^
	20–40	14.74 ± 0.97^c^	0.006 ± 0.001^c^
MNPK	0–20	16.21 ± 2.15^b^	0.007 ± 0.002^b^
	20–40	20.00 ± 1.31^b^	0.009 ± 0.002^b^
SNPK	0–20	22.51 ± 3.11^a^	0.010 ± 0.002^a^
	20–40	28.35 ± 4.19^a^	0.013 ± 0.003^a^
Source of variation			
Fertilization		**	**
Depth		*	*
Fertilization × Depth		n.s.	n.s.

The abbreviations for fertilization treatments are the same as presented in Table 1. Data are expressed as mean ± SE, n = 3. Different letters indicate statistical significance at *P* < 0.05 among fertilization treatments. Statistically significant differences are given after factorial ANOVA (n.s. not significant; *P < 0.05; **P < 0.01; ***P < 0.001).
